# Shedding the Last Layer: Mechanisms of Root Cap Cell Release

**DOI:** 10.3390/plants9030308

**Published:** 2020-03-01

**Authors:** Narender Kumar, Anjali S. Iyer-Pascuzzi

**Affiliations:** Department of Botany and Plant Pathology and Center for Plant Biology, Purdue University, West Lafayette, IN 47907, USA; kumar317@purdue.edu

**Keywords:** root cap, border cell, defense

## Abstract

The root cap, a small tissue at the tip of the root, protects the root from environmental stress and functions in gravity perception. To perform its functions, the position and size of the root cap remains stable throughout root growth. This occurs due to constant root cap cell turnover, in which the last layer of the root cap is released, and new root cap cells are produced. Cells in the last root cap layer are known as border cells or border-like cells, and have important functions in root protection against bacterial and fungal pathogens. Despite the importance of root cap cell release to root health and plant growth, the mechanisms regulating this phenomenon are not well understood. Recent work identified several factors including transcription factors, auxin, and small peptides with roles in the production and release of root cap cells. Here, we review the involvement of the known players in root cap cell release, compare the release of border-like cells and border cells, and discuss the importance of root cap cell release to root health and survival.

## 1. Introduction

**Figure 1:**
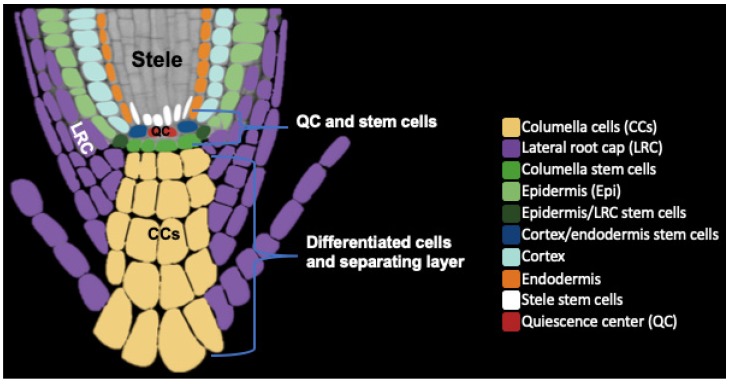
Schematic diagram showing different tissue organization of the root cap (CCs and LRC) and root apical meristem (QC and stem cells) regions. The last root cap layer is separating as a single intact layer known as BLCs (here colored purple and maize). Different cell types and tissues are colored differently.

Unlike animals, plant cells are fixed and attached together at the cell wall. However, there are several developmental processes in which cell separation is required and critical for plant survival. These include development of intercellular air spaces, leaf abscission, and seed dehiscence [1,2]. Another site of cell separation is the root cap, where the outer layer is sloughed off and released into the soil. The root cap is located at the tip of the root and protects the root apical meristem (RAM), a zone of dividing cells that produces all cells in the root (Figure 1). The root cap shields the RAM from abrasive damage and directs root growth by sensing environmental stimuli such as gravity, temperature gradients, water, touch, and chemicals [3,4,5,6].

Throughout root growth, the root cap maintains a constant size by coupling the production of new cells from root cap stem cells with the detachment of cells from the outermost layer of the root cap [6,7]. Root cap cell detachment results in a sleeve of loosely attached cells covering plant root tips. Because of their role at the root-soil interface, these cells are named “border cells (BCs)” [8,9,10]. In most plant species, BCs separate individually from the root cap and are viable after release from the root [10,11,12,13]. Studies in Arabidopsis, [14,15] and maize [11] have shown that BLCs and BCs can remain viable for days (Arabidopsis) to weeks (maize) after detachment. Unlike most other species, in Arabidopsis and other Brassicaceae members including *Brassica napus* (rapeseed), *Brassica oleracea* (Brussels sprout), and *Sinapis alba* (Mustard), BCs are released from the root tip as an organized layer of several cells that remain attached and are called “border-like cells (BLCs)” [10,14] (Figure 1). BLCs have also been reported in flax (*Linum usitatissimum*), a member of the Linaceae family [15]. BCs and BLCs protect the root from biotic and abiotic stress [8,12,15,16,17,18].

Root cap cell release is a developmentally programmed process that is linked with cell production and development. In this review, we will focus on molecular factors in the release of BLCs in Arabidopsis, comparisons of BLCs to BCs, and the contribution of root cap cell release to plant survival.

## 2. Root Cap Structure and BLC Release

The Arabidopsis root cap consists of several layers and is partitioned into two tissues: the columella (COL), at the tip of the root cap, and the lateral root cap (LRC) that surrounds the COL [19] (Figure 1). Both COL and LRC originate from distinct groups of stem cells located in the RAM. The RAM includes cortex/endodermis, stele, columella, and LRC/epidermis stem cells that surround a small group of infrequently dividing cells called the quiescent center (QC). COL stem cells divide asymmetrically to regenerate one stem cell and produce a daughter cell that undergoes differentiation and elongation without further division. The LRC originates from epidermis/LRC initials that are located laterally to the COL stem cells. LRC cells are located on the periphery of the COL and extend toward the shoot to the end of the root meristem. LRC cells undergo programmed cell death before their release [7]. Although some BCs and BLCs remain alive after separation [8,10,11,12,14,15], recent work suggests the presence of a programmed cell death mechanism active during COL cell separation [20]. The root cap secretes polysaccharide-containing mucilage that enhances the root’s ability to the move through the soil [21,22]. In young seedlings, a cuticle layer covers both the COL and LRC outer layers, but is not present at the root epidermis. This root cap cuticle acts as a protective barrier for the developing RAM and is observed primarily in one to three-day old seedlings and has mostly disappeared by 5 days, just before release of the first BLC layer [23]. The cuticle is present on the root caps of both primary and lateral roots, although the thickness and possibly molecular composition differ on each root cap [23].

In the first phase of root cap development, during the first four to five days after germination, the root cap grows through stem cell divisions without root cap cell release [24]. At the end of this first phase, the columella root cap has approximately five layers, including one stem cell tier, and four columella layers [24]. The first root cap layer is released once the Arabidopsis seedling is approximately five days old. At this point, coordination between stem cell division and separation of the outer root cap layer begins [24]. If columella stem cell activity is repressed, this will delay separation of the last layer [24]. Recent work used live imaging to reveal the temporal dynamics of root cap cell release [25]. Images were taken every 15 min for 64 h and 70 h, and revealed that root cap cell release is a multi-step process. In the first step, cell breaks occur in the LRC cell files just above the QC. Approximately 5 h later, another break occurs in the LRC around the youngest layer of COL cells. Finally, COL cells in the last root cap layer at the tip of the root separate from the root cap, and an intact layer of BLCs is released from the root [25]. The entire process takes an average of 18 h, and approximately 18 h passes prior to the initiation of the next release.

The release of BLC and BCs also depends on complex changes and rearrangements to cell wall polysaccharides, including the pectin homogalacturonan (HG) [26]. For example, in Arabidopsis, mutants defective in the synthesis of HG have altered BLC organization [27,28,29]. Additionally, in pea, the activity of pectin methylesterase (PME) is correlated with BC release [30,31], and inhibiting *PME* expression alters BC release [31].

To maintain a constant size and proper development, the root cap must manage regulation of cell division, cell differentiation, and the separation of the last layer. These processes are the outcome of coordination between transcriptional regulation, signal peptides, and hormone signaling. There has been progress in our understanding of the interplay between transcriptional factors and hormone signaling pathways during root cap development [32], and the relationship between root cap cell release and stem cell division is starting to emerge. In the following section, we will review small peptides and transcriptional regulation as well as hormone signaling in root cap development and BLC release.

## 3. Signaling Peptide and Transcriptional Regulation of BLC Release

**Figure 2:**
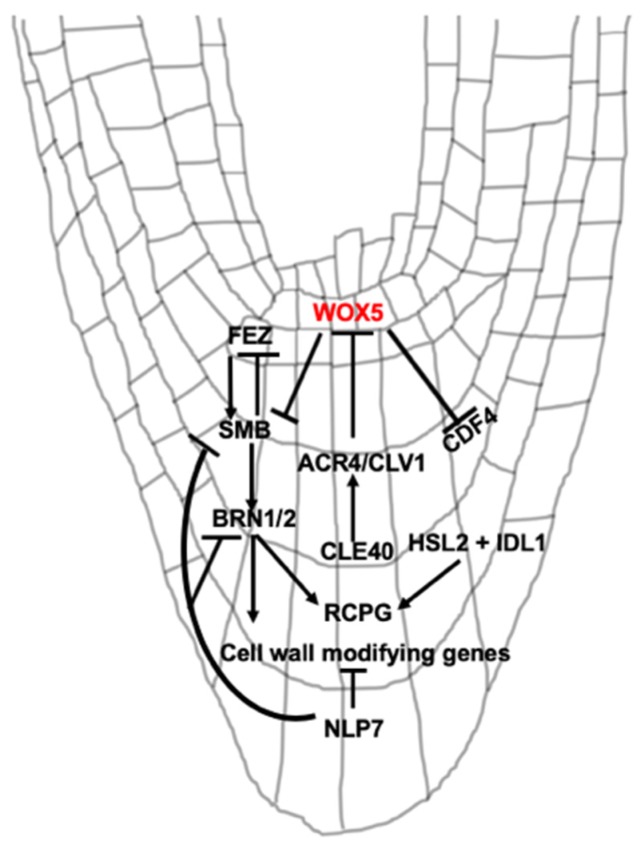
Involvement of different transcription factors and signaling peptides in Arabidopsis root cap development and BLC release. Arrows and barred lines indicate positive and negative regulation, respectively. Locations of proteins and peptides shown in the root are approximate.

Transcription factors and signal peptides are major players in BLC release (Figure 2). Because of the importance of root cap cell production to root cap cell release, we briefly review the transcription factors and peptides involved in cell production. For more extensive review, the reader is directed to [32]. For recent reviews of root apical meristem development and the maintenance of the root stem cell niche, see [33,34,35].

New COL cells are produced by COL stem cells (CSCs), which form one or two layers distal to the QC (Figure 1). CSCs remain undifferentiated, while stem cell daughter cells differentiate into COL cells. Differentiation is observed by the presence of starch granules, which are used for gravity perception. The balance between CSCs and differentiated COL cells is accomplished by the antagonistic actions of pathways that either promote ‘stemness’ or promote differentiation. The homeodomain transcription factor *WUSCHEL HOMEOBOX 5* (*WOX5*) is expressed in the QC where it suppresses QC cell division [36] and promotes the undifferentiated status of CSCs [37] (Figure 2). Mutations in *WOX5* lead to differentiation of CSCs, while overexpression increases the number of CSC layers [37,38]. Differentiated COL cells produce the small signaling peptide CLE40 [39]. CLE40 is perceived by the receptor kinases ARABIDOPSIS CRINKLY4 (ACR4) and CLAVATA1 (CLV1) which promotes COL cell differentiation [40] and the position of the QC [41]. The CLE40 module may regulate the movement of factors from the QC that promote ‘stemness’ [40,42]. Mutations in *CLE40*, *CLV1*, and *ACR4* increase CSC layers, while exogenous treatment of wild-type roots with CLE40 induces differentiation of CSCs [40]. In addition, another transcription factor, *CYCLIN DOF FACTOR4 (CDF4)* is expressed in COL cells and promotes their differentiation [43]. In CSCs, WOX5 represses expression of *CDF4* via histone deacetylation [43], which again promotes ‘stemness’. Other factors acting independently of *WOX5* also control the fate of CSCs [38,41,42].

Another signaling pair, the peptide IDA-LIKE1 (IDL1) and the receptor-like kinase HAESA-LIKE2 (HSL2), appears to coordinate the dynamics of root separation and production of new layers [25] (Figure 2). IDL1 is highly expressed in the COL, particularly in the last two layers. HSL2 expression is present in young LRC cells as well as upper tiers of the COL [25]. Overexpression of IDL1 in the root cap increased the frequency of BLC release, and this increased separation was compensated by the production of new COL cells [25]. How this signaling pair communicates to regulate the generation and separation of root cap layers is not fully clear.

Several NAC [NAC-domain stands for *N*AM (no apical meristem), *A*TAF, *C*UC (cup-shaped cotyledon)] transcription factors including *FEZ, SOMBRERO (SMB), BEARSKIN1 (BRN1), BRN2, ANAC046*, and *ANAC087* have important roles in root cap development and cell release. *FEZ* is preferentially expressed in root cap stem cells and promotes periclinal divisions in these cells [44] (Figure 2). FEZ activates expression of *SMB*. *SMB* subsequently acts to repress *FEZ* in stem cell daughters, preventing additional stem cell-like divisions in these cells [44]. The *fez* mutant has reduced COL and LRC layers [44]. *SMB* is expressed in differentiated COL and LRC layers. SMB has been implicated in the differentiation, maturation, and cell death of LRC cells [7,44,45]. Mutations in *SMB* lead to additional cell divisions in the root cap [44] delayed differentiation [45] and LRC cell death [7] and defective root cap cell detachment [45]. In addition, to maintain CSCs, WOX5 appears to exclude *SMB* expression from the CSCs [38] (Figure 2).

SMB modifies the spatial expression of *BRN1* and *2* [46], NAC-domain transcription factors that are important for root cap development. BRN1 and 2 function in cellular differentiation, maturation, and release of BLCs [38,45,46]. Expression of *BRN1* and *2* overlaps and is found primarily in the outer layers of the root cap, suggesting a role in the later phase of differentiation [46]. *BRN1* and *2* are also involved in mucilage accumulation [21]. BRN1 and 2, along with SMB, regulate the expression of genes associated with root cap differentiation and release of BLCs [45,46]. For example, the expression of cell wall degrading enzymes *CELLULASE3 (CEL3)* and *CEL5* is reduced in *smb brn1 brn2* triple mutants [45]. Further, *BRN1* and *2* expression overlaps with *ROOT CAP POLYGALACTURONASE (RCPG)*. RCPG is localized to the apoplast and is predicted to have a role in the degradation of cell wall pectins [46]. Overexpression of *RCPG* releases BLCs as individual cells, unlike a layer in wild-type Arabidopsis. *RCPG* is a direct target and activated by BRN1 (Figure 2) and *RCPG* expression is reduced in the *brn1 brn2* double mutant. Together, these data suggest a role of BRN1, BRN2, and RCPG in the separation of BLCs [46].

In addition to NAC transcription factors, the transcription factor *NIN-LIKE PROTEIN 7 (NLP7*) functions in release of BLCs (Figure 2). The *nlp7* mutant root releases a higher number of BLCs as single cells compared to wild-type plants [47]. Expression of *SMB, BRN1* and *2*, and cell wall loosening enzymes such as *CEL5*, a pectin lyase-like gene, and *XYLOGLUCAN ENDOTRANSGLUCOSYLASE (XTH5)* are upregulated in *nlp7-1*. *NLP7* is expressed in multiple tissues in the root, including the root cap. In the root cap, *NLP7* expression is highest in the last layer [47]. NLP7 is a major regulator of nitrate signaling in plants [48,49], but the functional connections between its roles in nitrate signaling and root cap development are not clear. 

In the past few years, good progress has been made in understanding regulatory mechanisms of root cap development and release. It appears that an intricate transcriptional regulation maintains root cap stem cells, differentiation, and release of BLCs (Figure 2). However, several questions remain unanswered or require additional clarity, as discussed in future perspectives below.

## 4. The Role of Hormones in BLC Release

**Figure 3:**
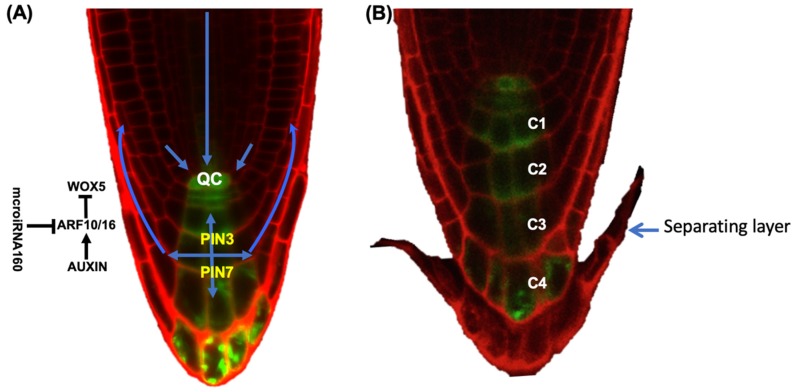
Auxin gradient in five-day old Arabidopsis root cap. (**A**) Columella localized PIN-FORMED (PIN) 3 and 7 and their flux direction. Auxin responsive factors (ARFs) 10/16 influence WOX5 to control columella cell differentiation and are regulated by auxin and miR160. Blue arrows indicate the direction of auxin flux. PIN3 and PIN7 are critical for auxin flux in the columella root cap, but additional PIN auxin transporters are required for flux in other cell types. Arrows and barred lines indicate positive and negative regulation, respectively. (**B**) The auxin response disappears from the separating layer. Auxin responsive synthetic promoter (pDR5:GFP) is used to show auxin response (green color). Columella (C) layers from C1 to C4. Arrows and barred lines indicate positive and negative regulations, respectively. Propidium iodide (red color) is used to visualize cells.

The hormone auxin functions as an instructive signal to direct transcriptional events that lead to changes in cell division, differentiation, and morphogenesis. It is an essential hormone in establishing and maintaining the root meristem. A gradient of auxin in the COL root cap is critical to BLC release and proper root cap development [24]. Before root cap cell release initiates, auxin responses are higher in the outer root cap layer compared to inner layers. However, at root cap cell separation, auxin response levels in the outer root cap layer become very low, and a sharp decrease is found in the layer of newly separated BLCs [24] (Figure 3). Thus, root cap cell separation occurs at auxin response minima. Consistent with a requirement for auxin minima for root cap cell separation, expression of the bacterial auxin biosynthesis gene *iaaM* in the outermost root cap layer increased the number of attached columella layers [24]. These data suggest that auxin response levels act as a threshold below which BLC separation occurs. The number of COL layers, COL length and the duration of the CSC division-BLC release cycle are dependent on the concentration of auxin in the QC and last layer [24]. Consistent with a role for auxin in the regulation of BLC release, auxin biosynthesis mutants have a lower rate of division in the CSCs and separation of the last layer, and a reduced columella size.

The COL expressed auxin efflux transporters PIN-FORMED3 (PIN3), 4 and 7 are important for auxin homeostasis in the root cap [50,51]. PINs can actively transport auxin against a concentration gradient to generate auxin maxima. However, modeling suggested that the root cap auxin gradient is largely the result of auxin movement, decay and dilution from cell elongation and release rather than from PIN transporters actively concentrating auxin in specific cells [24]. *PIN* expression patterns are important, though, as PINs are repressed in the fourth COL layer, which isolates the fifth separating layer from auxin and likely reinforces the auxin minimum [24] (Figure 3).

Auxin also regulates root cap development through the auxin transcription factors Auxin Response Factor10 (ARF10) and ARF16. ARF10 and ARF16 repress *WOX5* and limit it to the QC [52] (Figure 3). An increased number of cell divisions in the root cap stem cell area was observed in *arf10/16* double mutants [53]. In addition, the *arf10/16 smb* triple mutant has an additive effect on the number of root cap cell divisions [38]. Together, these data suggest roles for ARFs 10/16 in root cap differentiation, although whether ARF10/16 function in the release of BLCs is not known. ARFs 10/16 are also regulated by microRNA160 (miR160), an auxin-independent negative regulator of auxin response factors [53,54] (Figure 3).

## 5. Variations in BC Release Among Species

Release of BCs as individual cells, groups of cells or as a layer appears to depend on the organization of the RAM. RAM organization is conserved within taxa and organized in dicots as open, intermediate-open, and closed patterns based on the tiers of stem initials [55]. For example, *Arabidopsis thaliana* has closed organization in which all stem cell initials are arranged in the clonally distinct tiers. Carrot (*Daucus carota*) has an intermediate-open organization where root cap, cortex and vascular tissue originate from the same stem cell. Comparatively, in pea (*Pisum sativum*), the open RAM organization lacks clonally distinct tiers of stem cells, and different cell types cannot be traced back to specific types of stem cells. Species with open RAM organization produce significantly more BCs compared to species with a closed RAM organization [55]. The number of BCs is conserved at a family level, which can be from a few hundred (Solanaceae) to thousands (Fabaceae, Poaceae, Cucurbitaceae) to nearly 10,000 or more (Pinaceae and cotton) [13,17,55].

The morphology of released BCs also varies from species to species. BLCs released from Arabidopsis are spherical and elongated, while those released from flax are spherical, filamentous, and elongated even though both have a similar type of BLC (i.e., a single layer) [15]. Pea (*Pisum sativum*) releases BCs with a curved shape. Most are moderately curved (up to 45°), but others are nearly circular [26]. The curvature may be due to the maturation and detachment process, in which BCs elongate and subsequently curve away from the root cap surface. This curvature process is the consequence of complex modifications in BC cell wall components, including changes in pectin and in the distribution of xyloglucans (XyG) and extensin epitopes [26]. In separating BCs, XyG and extensin epitopes were polarly distributed such that they were present on the cell wall facing away from the root [26]. XyG and extension may be important for cell wall rigidity, and the authors speculate that their polar distribution in separating pea BCs helps promotes the BC curvature process [26]. 

*Brachypodium distachyon*, a model for grasses, has homogeneous and less curved border cells compared to pea [56]. Recent work suggests that spatial modifications to the pectin homogalacturonan during BC release are similar in *B. distachyon* and pea [56]. 

*Solanum tuberosum* releases two types of BC populations, one near the root tip known as “small border cells” and “elongated border cells” near the elongation zone. These BCs are viable after their detachment from the root tip [57]. Whether differences in BC number or morphology are functionally important is not clear.

## 6. BC and BLC Protective Functions in Biotic and Abiotic Stresses

The root cap assists the root in making a path through compact soil by secreting mucilage and protecting the RAM, which is vulnerable to damage and infection as it moves through dense microbial communities in the soil. BC and BLC release may act as the first point of defense for plant roots (recently reviewed in [18]) BCs protect the continuously growing root tip by releasing compounds such as protein, polysaccharides, or phytoalexins [18,57,58,59,60] and extracellular DNA in the rhizosphere [16,61,62]. Moreover, BCs are critical to root growth and plant health because these cells contribute to the nutrient-rich exudates in the vicinity of a root and may influence the microbial population by altering the physical and chemical properties of soil [12,16,18,63,64].

BCs from different plant species appear to provide a barrier against pathogens. For example, bacterial [flagellin22 (flg22) and peptidoglycan] and fungal (chitin and fusaric acid) microbe-associated molecular patterns (MAMPs) activate defense responses by triggering the production of reactive oxygen species in BLCs of both Arabidopsis and flax [15]. After elicitation with MAMPs, callose, a well-known cellular marker of plant defense, was observed in BLCs of both species, suggesting active defense responses in these cells [15]. In response to flg22 and the soilborne pathogen *Ralstonia solanacaerum,* pea (*Pisum sativum*) border cells release extracellular DNA which entangles microbes [62]. Mutating the DNase-coding gene in *R. solanacaerum* and the fungal pathogen *Cochliobolus heterostrophus* results in reduced virulence of these microbes [62,65].

Metabolites secreted from BCs also may function in defense. For example, flavonoid 7,4′-dihydroxyflavone from *Medicago truncatula* BCs inhibits growth of the soilborne fungal pathogen *Phymatotrichopsis omnivora*, causal agent of cotton root rot [66]. Exudates from pea root caps inoculated with *Nectria haematococca* suppressed growth of the fungus [64].

Arabinogalactan proteins (AGPs) are secreted from the cell walls of BCs and BLCs of several species [14,26,67] and may be involved in early events of root infection. For example, zoospores from *Aphanomyces euteiches*, an oomycete pathogen of pea, were attracted to pea AGPs in a chemotaxis assay [67]. In addition, germination of *A. euteiches* zoospores appeared to be inhibited by pea AGPs [67]. Potato (*Solanum tuberosum*) root exudate, enriched in galactose-containing molecules including arabinogalactan proteins, impacts the growth of bacteria *Pectobacterium atrosepticum* that causes stem wilting of potatoes [57].

Besides protection from biotic stresses, BCs have been reported to protect plant roots from abiotic stress. For example, BCs of different crops immobilize aluminum in order to protect the growing root [22,68,69]. In soybean (*Glycine max* L.), exposure to Al for 48 h promoted mucilage production in BCs [22]. Compared to BCs from an Al-sensitive cultivar, 48 h after exposure to 400 µM Al, BCs from an Al-resistant cultivar had thicker mucilage. Mucilage from Al-resistant soybeans bound more Al compared to that of Al-susceptible [22]. At high concentrations of Al (400 µM), BCs from the Al-resistant cultivar had higher viability compared to those from the Al-sensitive cultivar [22]. In snapbean (*Phaseolus vulgaris*), the viability of Al-resistant border cells exposed to 200 µM Al in an agarose gel was higher than that of border cells from an Al-susceptible cultivar [70]. In pea, Al bound to the alkali-soluble pectin in cell walls of BCs, which may protect the root tip from Al stress [69]. High levels of Fe^2+^ toxicity result in decreased border cell viability in rice [71]. Compared to a more Fe^2+^ tolerant rice genotype, Fe^2+^-sensitive rice roots had fewer BCs after exposure to high levels of Fe^2+^ [71].

## 7. Conclusions and Future Perspectives

Root cap cell release is developmentally programmed and influenced by transcription factors, small peptides, and hormones. While we are beginning to understand the molecular players involved in this process, many questions remain, including:

1. How do external stresses impact the release of root cap cells and the molecular pathways that control release? How are such stresses communicated in the root cap such that root cap cell production and release remain linked during environmental stress?
2. Do differences in either the timing or type (BLC vs BC) of release impact whole root growth and root architecture? Do BLC and BC release impact root cap protection differently?
3. How are signals between the separating layer and stem cells communicated and what are additional signals?
4. What are the mechanisms used by the auxin pathway to regulate cell production from CSCs and the release of BLCs?
5. What other transcription factors are involved in BLC and BC release? Are there other hormones besides auxin involved in BLC release? Does WOX5 have an impact on BLC release?
